# DipTest: A litmus test for *E*. *coli* detection in water

**DOI:** 10.1371/journal.pone.0183234

**Published:** 2017-09-06

**Authors:** Naga Siva Kumar Gunda, Saumyadeb Dasgupta, Sushanta K. Mitra

**Affiliations:** Micro & Nano-scale Transport Laboratory, Lassonde School of Engineering, York University, Toronto, M3J 1P3, Ontario, Canada; Institute of Materials Science, GERMANY

## Abstract

We have developed a new litmus paper test (DipTest) for detecting *Escherichia coli* (*E*. *coli*) in water samples by performing enzymatic reactions directly on the porous paper substrate. The paper strip consists of a long narrow piece of cellulose blotting paper coated with chemoattractant (at bottom edge), wax hydrophobic barrier (at the top edge), and custom formulated chemical reagents (at reaction zone immediately below the wax hydrophobic barrier). When the paper strip is dipped in water, *E*. *coli* in the water sample is attracted toward the paper strip due to a chemotaxic mechanism followed by the ascent along the paper strip toward the reaction zone due to a capillary wicking mechanism, and finally the capillary motion is arrested at the top edge of the paper strip by the hydrophobic barrier. The *E*. *coli* concentrated at the reaction zone of the paper strip will react with custom formulated chemical reagents to produce a pinkish-red color. Such a color change on the paper strip when dipped into water samples indicates the presence of *E*. *coli* contamination in potable water. The performance of the DipTest device has been checked with different known concentrations of *E*. *coli* contaminated water samples using different dip and wait times. The DipTest device has also been tested with different interfering bacteria and chemical contaminants. It has been observed that the different interfering contaminants do not have any impact on the DipTest, and it can become a potential solution for screening water samples for *E*. *coli* contamination at the point of source.

## Introduction

A litmus test is an indicative test that is used in chemistry to find the general acidity or alkalinity of the substance (liquid or gas) using litmus paper. A litmus paper is made of a dye based on lichens and it turns pink or red in an acid (pH < 6.0) whereas it turns blue in a base (pH > 8.0). There will be no color change in a solution with pH between 6.0 and 8.0. Litmus paper is inexpensive and is used to differentiate acids and bases. Similar kind of inexpensive litmus tests is not available in biology to identify or detect the biomolecules of interest for samples being tested. In the present work, for the first time, we have developed a litmus test using Whatman blotting paper that can be used to detect the *E*. *coli* bacteria in water samples. This kind of litmus paper strips are very useful in water quality testing to determine whether the water being tested is safe to drink or not.

Recent developments in paper-based biosensing technology have opened up an era of creating simple and low-cost rapid detection devices [1–6]. Most of the paper-based biosensors use the antigen-antibody interactions to detect the target analytes of interest in water, soil, urine, blood or saliva samples [3–6]. Applications built on paper based sensing technology are numerous ranging from testing of blood samples for infectious diseases, testing of grains in agriculture to testing of chemical contaminants in water and soil [1–6]. Hossain et al. [7] developed a paper-based microfluidic device to detect presence/absence of bacteria using chromogenic substrates. The bacteria in water samples is pre-concentrated using antibody-coated immune-magnetic nanoparticles and then tested the concentrated samples with the paper-based microfluidic device. They detected 5–20 CFU/mL within 30 min using a paper-based system without culturing step and then detected 1 CFU/100mL in 8 hrs with a culturing step. The use of nanoparticles and culture steps bring the complexity of the detection method. Ma et al. [8] developed an immunoassay based paper chips for detecting bacteria in water distribution system. Paper chips used for their work were fabricated by patterning the structures with wax pencil drawing and screen printing method. Further, they implemented the sandwich immunoassay procedure on the patterned areas for detecting *E*. *coli* bacteria. The use of antibody immobilization, blocking, immunology reaction and signal amplifications steps bring the complexity of the detection system. Recently, Silver Lake Research Corporation (Azusa, CA, USA) released a product, Watersafe rapid bacteria test, that detects *E*. *coli* in water samples within 15 minutes. The product is based on antigen-antibody interaction on paper strips similar to lateral flow tests. Water quality is evaluated by dipping the paper strip in contaminated water. The formation of two color bands on the paper strip represents the existence of *E*. *coli* in water samples. Even though these paper strips are simple to use, inexpensive and rapid but they are not specific to *E*. *coli*, fecal, or total coliform, and detects other non-coliform bacteria too. Table 1 provides a list of such existing commercial system and other relevant research work in context of detection of *E*.*coli* in water samples.

**Table 1:** Existing paper based test strips for the detection of *E*. *coli*. The commercial product is denoted by “*”.

Product/reference	Method	Sample Preparation	Specificity	Concentration (CFU/mL)	Time	Temperature	Type
Hossain et al. [7]	Paper strip coated with chromogenic substrate using silica gels and tested with pre-concentrated bacteria	Yes (pre-concentrated using antibody-coated immune-magnetic nanoparticles)	Yes	5 to 10^6^	30 min to 8 hr	Room temperature	Yes/No
Ma et al. [8]	Paper strips fabricated by drawing the patterns with wax pencil and screen printing method. Immunoassay method is applied for detection	Yes	Yes	10 to 10^6^	55 min	Not mentioned	Yes/No
Water Safe* from Silver Lake Research Corporation (Azusa, CA, USA)	Lateral flow test: antigen—antibody interaction on paper strip	No	No	Not mentioned	15 min	Room temperature	Yes/No
DipTest (similar to litmus paper)	Paper strip coated with chemoattractant and enzymatic substrate	No	Yes	200 to 10^5^	75 min to 3 hr	Room temperature	Yes/No

In the present work, for the first time, we have developed a simple and low-cost novel paper strip, similar to litmus paper, that can detect *E*. *coli* bacteria in water samples. The paper strip is made of a Grade GB003, Whatman absorbing gel blotting paper with one edge of the strip coated with wax hydrophobic barrier and the opposite edge (attraction zone) coated with D-glucose (dextrose) solution. There is a reaction zone immediately below the hydrophobic barrier on the paper strip, and it is coated with custom formulated chemical reagents comprising of red color producing Red-Gal substrate (6-Chloro-3-indolyl-*β*-D-galactoside), bacterial enzyme (protein) extracting reagent (B-PER) and nutrient medium Lauryl Tryptose Broth (LTB). It is to be noted that both the attraction and reaction zones are present on single paper itself. The D-glucose coated edge of the novel paper strip needs to be dipped into the contaminated water for detecting *E*. *coli* bacteria. D-glucose acts as a chemoattractant to attract the bacteria in water samples towards the paper strip [9] and then water along with attracted bacteria percolates through porous network of the paper strip towards the reaction zone by capillary action and then stops at the wax hydrophobic barrier. The use of blotting paper allowed a uniform capillary movement of water along with bacteria towards reaction zone without any requirement for additional pumps. Since this novel paper strip is dipped into water and tested for bacteria, we call this paper strip as DipTest device [10, 11], and now onwards, this paper strip is mentioned as DipTest in this article. The current DipTest device is simple and easy to use compared to the currently available water testing kits in the market and also with the previously developed portable water testing kits (Mobile Water Kit (MWK) [12–14] and plunger-tube assemblies [15–17]) developed by our group.

## Materials and methods

### Materials

Whatman gel blotting paper (0.8 mm thickness, Grade GB003), enzymatic substrate Red-Gal (6-chloro-3-indolyl-*β*-D-galactoside) and N, N-Dimethylformamide (DMF) were procured from Sigma Aldrich, Canada. Lauryl Tryptose Broth (LTB) (BD 224150), Bacteria protein extraction reagent (B-PER), Veal Infusion Broth (BD 234420), Bacto Yeast Extract (BD 212750), Brain Heart Infusion Broth (BD 237500), and Nutrient Broth (BD 234000) were purchased from Fisher Scientific, Canada.

Bacteria strains such as *E*.*coli* Castellani and Chalmers (American Type Culture Collection (ATCC) 11229), *Enterococcus faecalis* (*E*.*faecalis*) (ATCC 19433), *Salmonella enterica subsp*. *enterica* (*S*.*enterica*) (ATCC 14028) and *Bacillus subtilis* (*B*.*substilis*) (ATCC 33712, MI112 strain) were obtained from Cedarlane, Burlington, ON, Canada. *E*.*coli* K-12 strains were purchased from New England Biolabs, Ipswich, Massachusetts, USA.

*E*.*coli* ATCC 11229 and *E*.*coli* K-12 were grown in LTB medium as well as in nutrient broth medium at 37°*C* in incubator (Lab Companion SI-300 Benchtop Incubator and Shaker, GMI, Ramsey, Minnesota, USA) for 24 hours. *B*.*subtilis* bacteria strains were cultured in a growth medium consisting of Veal Infusion Broth and Yeast Extract (5:1 ratio) at 30°C in incubator for 24 hrs whereas *E*.*faecalis* and *S*.*enterica* were grown in brain heart infusion broth medium and nutrient broth medium, respectively. Deionized (DI) water was used to prepare the respective broth medium. Broths were sterilized in an autoclave at 121°*C* prior to using them for culturing the respective bacteria. Serial dilutions were prepared in DI water to make bacteria concentrations in the range of 2–2 × 10^6^ CFU/mL. Water samples with known concentrations of bacteria were utilized to check the performance of DipTest device.

Sodium fluoride, EMD ferric chloride (hexahydrate), and EMD sodium chloride were procured from Fisher Scientific, Canada. Sodium nitrate, iron Chloride hexahydrate, ammonia persulfate, sodium iodide, sodium sulfate, potassium hydroxide, sodium bromide, sodium phosphate, and calcium propionate were purchased from Sigma Aldrich, Canada. Standard fluoride solution (1ppm), fluoride solution (10ppm), cadmium and lead were obtained from Hanna instruments, Woonsocket, RI, USA.

### Methods

#### Preparation of custom formulated chemical composition

In the present work, we formulated a new chemical composition by dissolving 100 mg of solid media (1:1 mixture of LTB and Red-Gal) in 4 mL of liquid media (1:2:5 mixture of DMF, B-PER and DI water). The enzymatic substrate Red-Gal is used to detect *E*. *coli* that secrete *β*-galactosidase enzymes. A chromogenic compound Red-Gal (6-Chloro-3-indolyl-*β*-D-galactoside) contains two components: 6-Chloro-3-indolyl and *β*-D-galactoside. The *β*-galactosidase enzyme produced by *E*. *coli* hydrolyses this complex Red-Gal molecule resulting in the release of pinkish red color producing dimerized 6-Chloro-3-indolyl compound. The inclusion of B-PER in custom formulated chemical reagents is to accelerate the extraction of *β*-galactosidase enzymes by lysing the *E*. *coli* bacteria cells without denaturing the bacterial enzymes.

#### Preparation of DipTest device

**Fig 1:**
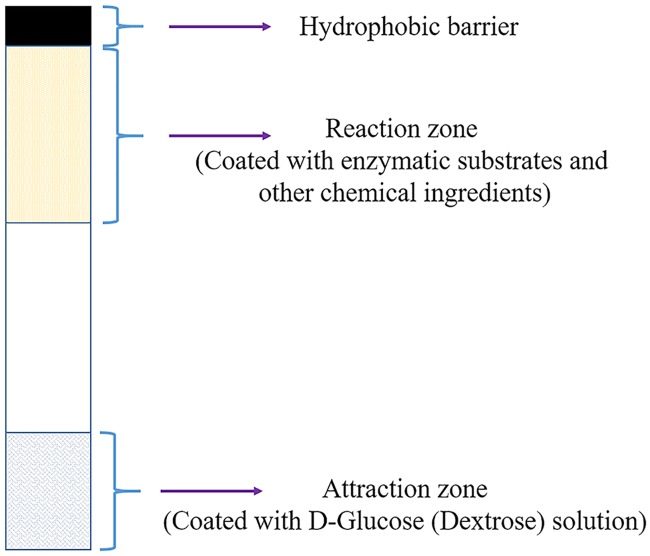
Schematic of the DipTest device.

Initially, the blotting paper is diced into 70 mm × 5 mm size strips. The length of paper strip chosen i.e. 70 mm is enough for the capillary imbibition to occur. Blotting paper is made of pure cellulose produced entirely from the high quality cotton linters with no additives. Blotting paper has a weight of 320 g/m^2^, wet strength of 300 mm water column and water absorbency of 740 g/m^2^. The blotting paper ensures the proper wicking and uniform capillary action. One edge of the paper strip is coated with wax to form a hydrophobic barrier. The wax barrier prevents the further spreading of the chemicals and bacteria in the reaction zone through capillary action. The reaction zone is formed below the hydrophobic barrier by depositing the 100 *μ*L of above mentioned custom formulated chemical composition (Red-Gal, B-PER and LTB) using pipette and followed by drying under normal laboratory condition (temperature around 23°C) for one hour. After coating custom formulated chemical composition at the reaction zone, the opposite edge of the paper strip is coated with D-glucose (dextrose) by dispensing 100 *μ*L of 0.1 M D-glucose and then allowed to be dried at room temperature (23°C) for one hour. This edge is also known as attraction zone since D-glucose acts as a chemotaxis agent to attract the bacteria towards the paper strip. The resulting paper strips were completely dried for one hour under a fume hood before dipping them into *E*. *coli* contaminated water. The schematic of the DipTest is provided in Fig 1.

#### Testing water samples with DipTest device

To perform the test, the edge with attraction zone of DipTest device needs to be dipped into the *E*. *coli* contaminated water. The D-glucose in the attraction zone gets dispersed and forms a concentration gradient in the water. This gradient creates the chemotactic movement of *E*. *coli* bacteria from the surrounding water and it eventually increases the migration of bacteria towards the paper strip [9]. The water along with bacteria (attracted at the edge of the paper strip) percolates into the porous matrix of paper strip due to capillary action. Once the water front reaches the hydrophobic barrier on a paper strip (DipTest), the DipTest is removed from the water and kept aside on a flat surface. The bacteria trapped in the reaction zone will react with chemicals and produce the pinkish red color. The appearance of pinkish red color indicates the presence of *E*. *coli* bacteria. It is to be noted that all the tests with DipTest are conducted at room temperature.

## Results and discussion

The present DipTest device uses a glucose at the attraction zone and a custom formulated chemical composition (LTB, B-PER and Red-Gal) at the reaction zone. Glucose allows the *E*. *coli* in contaminated water to reach the attraction zone whereas the custom formulated chemical composition allows the *E*. *coli* to interact and produce the color (pinkish red color appearance) at a reaction zone within minutes of testing contaminated water.

### DipTest device performance

**Fig 2:**
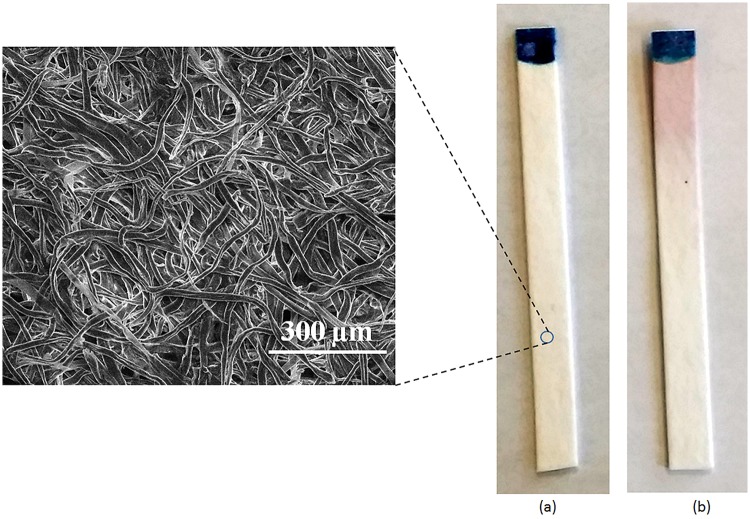
Comparison of the DipTest device between (a) tested with DI water at room temperature (inset shows the scanning electron microscope (SEM) image of porous paper matrix) (b) tested with *E*. *coli* contaminated water (2 × 10^4^ CFU/mL) at room temperature. It is to be noted that the appearance of color on the used DipTest device represents the presence of *E*. *coli*.

Fig 2 illustrates the color change at the reaction zone of DipTest device because of the presence of a known concentration of *E*. *coli* (ATCC11229) in contaminated water. It is observed that there is a pinkish red color at the reaction zone of the DipTest device, which represents the presence of *E*. *coli*. A controlled study is conducted where DipTest device was tested in DI water at room temperature with no *E*. *coli* and it is found that there is no color change in the reaction zone. The inset of Fig 2(a) shows the scanning electron microscope image of the porous paper matrix. It is observed that the paper is randomly distributed network of paper fibres with an estimated porosity of 65 to 73%.

**Fig 3:**
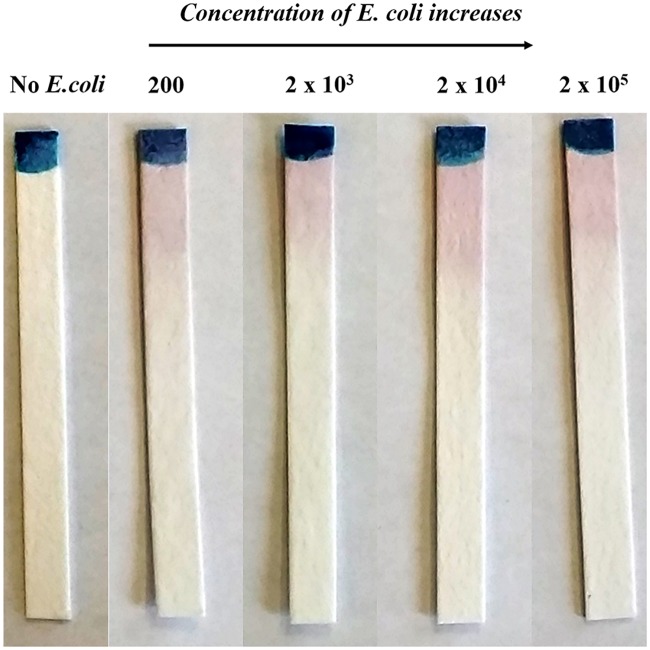
Development of pinkish red color on the DipTest device after 2 hrs based on the concentrations of *E*. *coli* (CFU/mL). The control strip is with DI water, which shows no color.

Fig 3 shows the appearance of pinkish red color at the reaction zone of the DipTest device for various known concentrations of *E*. *coli* (ATCC 11229) contaminated water samples after 2 hrs at room temperature. It is to be noted that the color intensity varies based on the concentration of bacteria in water samples and how much time the DipTest device is dipped into the water. It is found that the color intensity decreases with the decrease in the concentration of *E*. *coli*. This figure also shows the sensitivity and limit of detection of DipTest device. The present method could detect up to 200 CFU/mL and this can be considered as the limit of detection.

**Fig 4:**
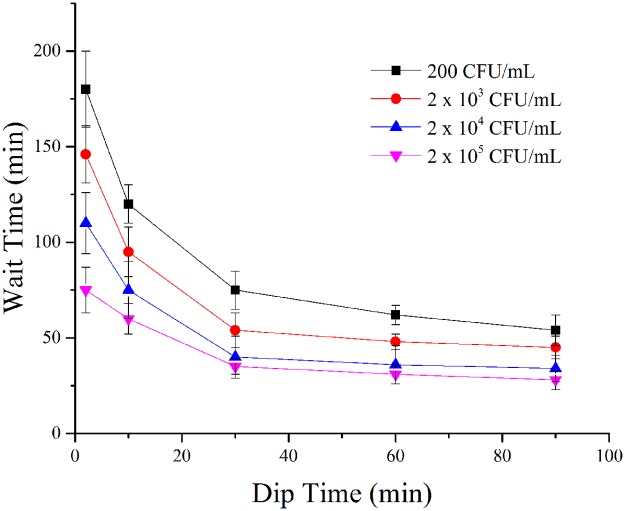
Comparison of DipTest wait (response) times for the appearance of the pinkish red color with respect to various dip times. The plot is shown for various known concentrations of *E*. *coli* spiked water samples.

The performance of the DipTest device is evaluated based on the dip time and wait time. Dip time is the amount of time the DipTest device is immersed in the water samples whereas wait time (response time) is the amount of time one has to wait for the results (appearance of pinkish red color) after removing the DipTest device from water samples. Fig 4 portrays the comparison of DipTest wait (response) times for the appearance of pinkish red color at reaction zone at various dip times and for different known concentrations of *E*. *coli* spiked water samples. The average wait times with error bars are provided in the Fig 4. It is observed that the appearance of pinkish red color at reaction zone of DipTest device for samples with 2 × 10^5^ CFU/mL to 4 × 10^4^ CFU/mL happens in 60 to 65 min (wait time) corresponding to a dip time of 2min. It is also observed that wait time decreases with the increase in dip times. The increase in dip time allows the more number of *E*. *coli* bacteria to accumulate at the reaction zone, which in turn decreases the wait time to produce the color due to presence of *E*. *coli* bacteria. It is also found that the lower concentrations of *E*. *coli* spiked water samples take more wait (response) times compared to higher concentrations of *E*. *coli*. The space between attraction zone and reaction zone will not influence the performance of the device as we are keeping the paper strip in water samples for longer dip times. However, the optimized length of paper strip is required to maintain the stability of the paper strip to sustain the water absorbency for longer time. The length of paper strip chosen here is based on that parameter.

The wicking of *E*. *coli* contaminated water into porous paper matrix follows the Washburn-Lucas equation and it is given as [18–23],

$$\begin{array}{c} L^{2}=\gamma Dt/4\eta^{*} \end{array}$$ (1)

where, *L* is the distance moved by the fluid front, *γ* is the effective surface tension (which includes the effect of any contact angle dependency), *D* is the average pore diameter of paper, *t* is the time and η* is the effective viscosity of *E*. *coli* contaminated water. Effective viscosity depends on the concentration of *E*. *coli* bacteria. The effective viscosity of *E*. *coli* contaminated water is provided as [24],

$$\begin{array}{c} \eta^{*}=\eta [1+2\phi -\frac{c}{2\pi \eta \epsilon_{o}}\frac{1+2\lambda (2+\lambda )}{{\left(1+\lambda \right)}^{3}}\phi ] \end{array}$$ (2)

where, *η* is the viscosity of water without *E*. *coli* bacteria, *ϕ* is volume fraction occupied by *E*. *coli* bacteria in water, *ϵ*_*o*_ is the amplitude of the strain rate, *c* is the point force representing the flagellum, *λ* is the length of the run between tumbles, representing bacteria motility. By neglecting the motility effects, one can obtain the effective viscosity of the *E*. *coli* contaminated water as

$$\begin{array}{c} \eta^{*}=\eta [1+2\phi ] \end{array}$$ (3)

*E*. *coli* bacteria are usually in rod-shaped and are about 0.25–1.0 *μ*m in diameter and 2.0 *μ*m long, with a bacterial volume of 0.6–0.7 *μ*m^3^ [25]. Based on the concentrations of bacteria (2 × 10^5^ CFU/mL to 200 CFU/mL) used in the present work, the volume fraction occupied by *E*. *coli* bacteria in water varies from 1.4 × 10^−7^ to 1.4 × 10^−10^, which in turn dictates that there is negligible effect of bacterial suspensions on the viscosity of the contaminated water. Therefore, for further analysis, one needs to decouple the hydrodynamic effects from the reaction kinetics responsible for the appearance of the pinkish red color on the paper strips.

The initial rate of interaction of Red-Gal substrate with *β*-galactosidase enzyme can be described by the Michaelis-Menten equation [26],

$$\begin{array}{c} \upsilon_{o}=\frac{k_{cat}E_{o}}{1+\frac{K_{m}}{S}} \end{array}$$ (4)

where, *k*_*cat*_ is turnover number and *E*_*o*_ is concentration of *β*-galactosidase enzyme (released from *E*. *coli* bacteria), *K*_*m*_ is Michaelis constant and *S* is the concentration of Red-Gal substrate. It is clear that the wait time for color appearance is solely depended on the interaction of the Red-Gal substrate with *β*-galactosidase enzyme. The presence of B-PER at the reaction zone helped to accelerate the production of *β*-galactosidase enzyme from *E*. *coli*.

### Effect of E. coli growth medium on DipTest performance

In order to study the effect of *E*. *coli* growth medium on performance of DipTest device, the two *E*. *coli* bacteria strains ATCC11229 and K-12 were grown in LTB medium as well as in nutrient broth medium. Water samples contaminated with these *E*. *coli* bacteria are tested with DipTest device. It is observed that DipTest device produced pinkish red color with both kind of samples. However, the *E*. *coli* bacteria cultured in LTB medium generated a high intensity color compared to the bacteria grown in nutrient broth medium.

### Effect of interfering bacteria and chemical contaminants

The DipTest device performance is verified for its specificity by testing the device with several water samples containing different interfering bacteria and chemical contaminants. Table 2 shows the DipTest results for 40 different water samples. Diptest device is tested with water samples containing several interfering bacteria. *B*.*subtilis*, *E*.*faecalis* and *S*.*enterica* were used as interfering bacteria. For Category A (Samples # 1–3) water samples, i.e., water samples containing only interfering bacteria (*B*.*subtilis*, *E*.*faecalis* or *S*.*enterica*) and without *E*. *coli* bacteria do not produce any color. On the other hand, Diptest device produces color for water samples that contain both interfering bacteria and *E*.*coli* (i.e., Category B Samples # 4–7). It is found that the interference bacteria have no effect on the detection of *E*.*coli* with DipTest device. It is to be noted that the custom formulated chemical composition developed in this work is specific to *E*. *coli* based strains. The chemical composition is favorable to the growth of *E*. *coli* and production of the *β*-galactosidase enzyme. The interfering species we have chosen in this work (*B*.*subtilis*, *E*.*faecalis* and *S*.*enterica*) do not produce the *β*-galactosidase enzyme with our chemical composition and in turn, do not react with Red-Gal to produce the color. It is also noted that all strains of *B*.*subtilis* and *S*.*enterica* do not produce the *β*-galactosidase enzyme. It is required to have favorable growth conditions to produce the *β*-galactosidase enzyme [27, 28]. For example, the *β*-galactosidase enzyme can be produced by the growth of selected *B*.*subtilis* strains at 10°C in a growth medium containing 2% (w/v) lactose supplemented with 0.2% (w/v) yeast extract [27, 28]. As the chemical composition used in this work is not a combination of these compounds, therefore, the interfering bacteria are not able to grow and produce the *β*-galactosidase enzyme. The custom formulated chemical composition coated on DipTest device is the main component which ensures the specificity to detect *E*. *coli*. The chemical composition contains the favorable growth medium i.e. Lauryl Tryptose Broth (LTB) and enzymatic substrate (Red-Gal). LTB favors the maximum possible growth of *E*. *coli* as well as the maximum possible production of the *β*-galactosidase enzyme. The produced *β*-galactosidase enzyme hydrolyzes the Red-Gal and thereby produces the pinkish red color.

**Table 2:** DipTest results for 40 different water samples used in this work.

Category	Sample No.	Contents in Water Samples	DipTest (Dip time = 90 min; Wait time = 180 min)
**A**	1	*B*.*subtilis*	No Color
	2	*E*.*facecalis*	No color
	3	*S*.*enterica*	No color
**B**	4	ATCC11229, *E*. *coli* K-12 and *B*.*subtilis*	Color produced
	5	ATCC11229, *E*. *coli* K-12 and *E*.*facecalis*	Color produced
	6	ATCC11229, *E*. *coli* K-12 and *S*.*enterica*	Color produced
	7	ATCC 11229, *E*. *coli* K-12, *E*.*facecalis*, *S*.*enterica*, and *B*.*subtilis*	Color produced (low intensity)
**C**	8	Sodium fluoride	No color
	9	Sodium nitrate	No color
	10	Iron Chloride hexahydrate (EMD)	No color (paper strip turned yellowish)
	11	Ammonia persulfate	No color (paper strip turned light yellowish)
	12	Sodium sulfate	No color
	13	Sodium bromide	No color
	14	Sodium iodide	No color
	15	Sodium phosphate	No color
	16	Ferric chloride, hexahydrate (Sigma)	No color (yellowish)
	17	Sodium chloride	No color
	18	Calcium propionate	No color
	19	Potassium hydroxide	No color
	20	Fluoride solution (1ppm)	No color
	21	Fluoride solution (10ppm)	No color
	22	Cadmium	No color
	23	Lead	No color
**D**	24	ATCC 11229, E.coli K-12, and sodium fluoride	Color produced
	25	ATCC 11229, E.coli K-12, and sodium nitrate	Color produced
	26	ATCC 11229, E.coli K-12, and iron chloride hexahydrate (EMD)	Color produced
	27	ATCC 11229, E.coli K-12, and ammonia persulfate	Color produced (high intensity)
	28	ATCC 11229, E.coli K-12, and sodium sulfate	Color produced
	29	ATCC 11229, E.coli K-12, and sodium bromide	Color produced
	30	ATCC 11229, E.coli K-12, and sodium iodide	Color produced
	31	ATCC 11229, E.coli K-12, and sodium phosphate	Color produced
	32	ATCC 11229, E.coli K-12, and Ferric chloride, hexahydrate (Sigma)	Color produced
	33	ATCC 11229, E.coli K-12, and sodium chloride	Color produced
	34	ATCC 11229, E.coli K-12, and calcium propionate	Color produced
	35	ATCC 11229, E.coli K-12, and potassium hydroxide	Color produced
	36	ATCC 11229, E.coli K-12, and fluoride solution (1ppm)	Color produced
	37	ATCC 11229, E.coli K-12, and fluoride solution (10ppm)	Color produced
	38	ATCC 11229, E.coli K-12, and cadmium	Color produced
	39	ATCC 11229, E.coli K-12, and lead	Color produced (low intensity)
**E**	40	DI water without bacteria and chemical contaminants	No Color

Similarly, we tested the DipTest device with water samples containing several kind of chemical contaminants with and without *E*.*coli* bacteria. DipTest device do not give any color when it is tested with water samples (Category C, water samples # 8–23) containing different chemical contaminants. However, DipTest device is able to produce the color (pinkish red color) when the device is tested with water samples containing *E*.*coli* along with different chemical contaminants (Category D, water samples # 24–39). It is clearly indicated that chemical contaminants do not react with the chemicals (Red-Gal, B-PER and LTB) coated on DipTest device and also they are not interfering with *E*.*coli* bacteria when they are interacting with chemicals (Red-Gal, B-PER and LTB) on DipTest device. Similarly, we tested the DipTest device with negative control i.e., DI water without having any bacteria and chemical contaminants (Category E Sample # 40). It is found that the pinkish red color is not produced on DipTest device for this negative control. It is clearly showing that the DipTest device is working fine under different kind of water samples for both positive and negative controls as well as with interfering bacteria and chemical contaminants.

### Future outlook: Towards a field deployable water testing device for bacterial contamination

**Fig 5:**
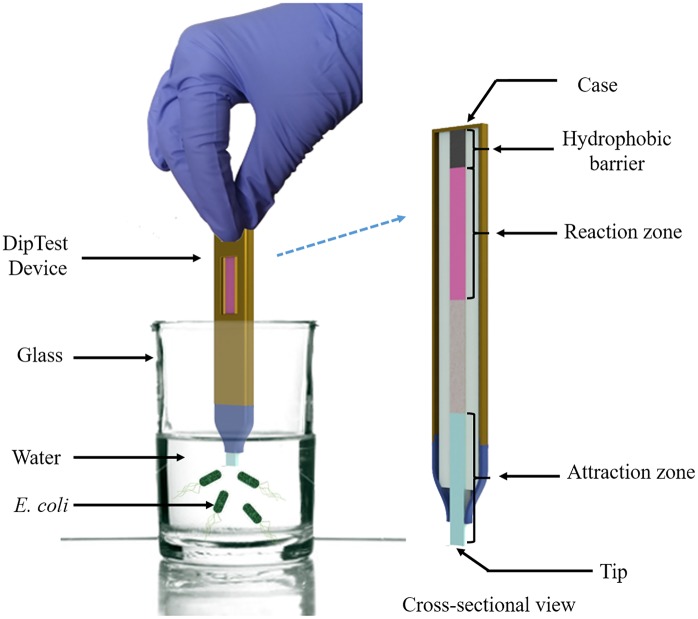
Representation of the use of DipTest device to test the water sample for the presence of *E*. *coli* bacteria.

Fig 5 shows the illustration of the use of DipTest device for the detection of *E*. *coli* bacteria in water samples. One has to dip the DipTest device in water for testing purpose. The device can be immersed in water for a certain time and then be removed from the water and placed on a flat surface for the result. DipTest device would be a litmus paper for determining whether the water is safe from bacterial contamination or not. This DipTest device is very useful in remote locations where one can dip this device and find whether the water is safe to use or not. In particular, it is very useful for checking the quality of water in swimming pools, lakes, rivers, and beaches.

## Conclusion

In summary, we have developed a novel paper based DipTest device, similar to a litmus test, for detection of *E*. *coli* bacteria in water samples. The DipTest device is easy to fabricate and simple to test the water samples. Currently, for a dip time of 2 min, DipTest device is able to detect as low as 200 CFU/mL in 180±20 min and higher concentrations such as 2 × 10^5^ CFU/mL within 75±12 min. However, for a dip time of 90 min, DipTest device is able to detect as low as 200 CFU/mL in 54±8 min and higher concentrations such as 2 × 10^5^ CFU/mL within 28±5 min. The performance of DipTest device is checked and verified under different kind of water samples containing interfering bacteria and chemical contaminants. This DipTest device would eventually act as a field screening test that can be carried in a pocket and one can conduct the testing of water samples whenever required. The DipTest device can also be disposed off easily after completion of test with minimal effort. Further optimizations in terms of the concentration of individual chemical ingredients used here are needed so that one can eventually have a field deployable device to provide “yes/no” litmus test for *E*. *coli* concentration as low as 1–10 CFU/100 mL, thereby meeting the US EPA standards [29]. The current DipTest platform can be adapted and integrated with further developments in the detection of other bacteria and pathogens and used not just for water samples but for many other products (milk, wine, juices, etc.) and food industry (frozen meat and cheese).
